# The Brazilian national prospective active surveillance (AS) cohort of patients with low-risk prostate cancer in the public health system: vigiaSUS study protocol

**DOI:** 10.1186/s12894-023-01380-w

**Published:** 2023-12-11

**Authors:** Jeziel Basso, Juliana Beust de Lima, Marina Bessel, Santiago Alonso Tobar Leitão, Thais Machado Baptista, Sergio Roithmann, Eduardo Franco Carvalhal, Caio da Silva Schmitt, Ivan Morzoletto Pedrollo, Alice Schuch, Antonio Atalibio Hartmann, Carmen Liane Neubarth Estivallet, Guilherme Behrend Silva Ribeiro, Ricardo Andre Zordan, Pedro Isaacsson Velho

**Affiliations:** 1 Oncology Department, Hospital Moinhos de Vento, Porto Alegre, Rio Grande do Sul Brazil; 2 Urology Department, Hospital Moinhos de Vento, Porto Alegre, Rio Grande do Sul Brazil; 3 Project Office, PROADI-SUS, Hospital Moinhos de Vento, Porto Alegre, Rio Grande do Sul Brazil; 4 Radiology Department, Hospital Moinhos de Vento, Porto Alegre, Rio Grande do Sul Brazil; 5 Pathology Department, Hospital Moinhos de Vento, Porto Alegre, Rio Grande do Sul Brazil; 6 Oncology Department, Johns Hopkins Sidney Kimmel Comprehensive Cancer Center, Baltimore, USA

**Keywords:** Active surveillance, Prostate cancer, Prospective cohort

## Abstract

**Background:**

Prostate cancer exhibits a very diverse behaviour, with some patients dying from the disease and others never needing treatment. Active surveillance (AS) consists of periodic PSA assessment (prostate-specific antigen), DRE (digital rectal examination) and periodic prostate biopsies. According to the main guidelines, AS is the preferred strategy for low-risk patients, to avoid or delay definitive treatment. However, concerns remain regarding its applicability in certain patient subgroups, such as African American men, who were underrepresented in the main cohorts. Brazil has a very racially diverse population, with 56.1% self-reporting as brown or black. The aim of this study is to evaluate and validate the AS strategy in low-risk prostate cancer patients following an AS protocol in the Brazilian public health system.

**Methods:**

This is a multicentre AS prospective cohort study that will include 200 patients from all regions of Brazil in the public health system. Patients with prostate adenocarcinoma and low-risk criteria, defined as clinical staging T1–T2a, Gleason score ≤ 6, and PSA < 10 ng/ml, will be enrolled. Archival prostate cancer tissue will be centrally reviewed. Patients enrolled in the study will follow the AS strategy, which involves PSA and physical examination every 6 months as well as multiparametric MRI (mpMRI) every two years and prostate biopsy at month 12 and then every two years. The primary objective is to evaluate the reclassification rate at 12 months, and secondary objectives include determining the treatment-free survival rate, metastasis-free survival, and specific and overall survival. Exploratory objectives include the evaluation of quality of life and anxiety, the impact of PTEN loss and the economic impact of AS on the Brazilian public health system.

**Discussion:**

This is the first Brazilian prospective study of patients with low-risk prostate cancer under AS. To our knowledge, this is one of the largest AS study cohort with a majority of nonwhite patients. We believe that this study is an opportunity to better understand the outcomes of AS in populations underrepresented in studies. Based on these data, an AS national clinical guideline will be developed, which may have a beneficial impact on the quality of life of patients and on public health.

**Trial registration:**

Clinicaltrials registration is NCT05343936.

**Supplementary Information:**

The online version contains supplementary material available at 10.1186/s12894-023-01380-w.

## Background

Prostate cancer is the most common cancer in men. The Instituto Nacional de Câncer (INCA) estimated 65,840 new cases of prostate cancer in Brazil for each year of the 2020–2022 triennium, which represents 29.2% of new cases of cancer in men in Brazil [1].

Prostate cancer has a very diverse behaviour, with some patients dying from the disease and others never needing treatment. PSA (prostate-specific antigen), digital rectal examination (DRE) and Gleason score are used to classify patients into different risk groups, which helps in treatment decisions. Low-risk prostate cancer is defined as clinical staging cT1- T2a, Gleason score ≤ 6 on tumour biopsy and PSA less than 10 ng/ml [2].

Historically, localized prostate cancer has been managed with radical treatments such as prostatectomy and radiotherapy. These interventions can result in potentially serious adverse effects, especially urinary incontinence and erectile dysfunction [3, 4]. Active surveillance consists of periodic assessments with PSA, digital rectal examination and periodic prostate biopsies [5]. AS is the preferred strategy for low-risk patients to avoid or delay definitive treatment. Results in cohorts of patients on AS demonstrated that this strategy is safe in low-risk patients and is considered standard of care by the main guidelines [6–11].

Despite progress made in the use of AS as a treatment option for prostate cancer, concerns remain regarding its applicability in certain patient subgroups, such as African American (AA) men, who are known to have worse prostate cancer outcomes [12]. Research has shown that African American men were notably underrepresented in the main cohorts that demonstrated favourable outcomes in low-risk prostate cancer. Furthermore, these men often have less access to treatments than Caucasian American men, which can lead to disparities in outcomes [13]. In Brazil, where the population is composed of a diverse mix of races, 56.1% of individuals self-report as brown or black according to IBGE data [14].

AS clinical outcomes and economic impact have never been evaluated in Brazil or in the Brazilian public health system. Therefore, this study seeks to validate the AS strategy in the Brazilian population by evaluating an AS cohort of patients and demonstrate the viability of the strategy in the public system. Based on these data, an AS national clinical guideline will be proposed, which may have a beneficial impact on the quality of life of patients and on public health expenditures.

### General objective

To evaluate the outcomes in a cohort of patients with low-risk localized prostate cancer followed by an AS protocol in specialized centres in the Brazilian public health system.

### Specific objectives

In patients with low-risk prostate cancer undergoing AS, to evaluate the following:

- Pathological reclassification rate at 12 months.
- Treatment-free survival rate.
- Prostate cancer-specific mortality and overall mortality.
- Metastasis-free survival.
- Quality of life through EPIC-CP and EQ-5D-5 L questionnaires.
- Anxiety symptoms, using the GAD-7 questionnaire.
- An economic impact analysis of the AS strategy.
- The perception of urologists regarding the AS strategy in patients with low-risk localized prostate cancer in the Brazilian public health system.

### Translational analysis

- To evaluate whether PTEN loss by immunohistochemistry has an impact on the clinical outcomes of low-risk prostate cancer patients on AS.

## Methods

### Design

The VigiaSUS study is a multicentre, nationwide, prospective cohort study.

### Recruitment

The study will include patients from all 5 regions of Brazil. Centres will be selected based on the high recruitment potential, experience in the treatment of localized prostate cancer and availability of multiparametric MRI. The centres must agree to send biological samples from prostatic biopsies for diagnostic review and agree to use an AS protocol as a guide for monitoring low-risk prostate cancer. The inclusion of patients in all regions of the country will be representative of the national population. All eligible individuals who meet the inclusion criteria to participate in the study will be considered and invited.

### Inclusion criteria

Eligible patients must be between 18 and 78 years old, have a localized prostate adenocarcinoma diagnosis within 12 months of inclusion and have low-risk disease. Diagnostic prostate biopsy must have at least 12 cores and Gleason score below or equal to 6 (3 + 3). Patients must have PSA less than or equal to 10 ng/ml and clinical staging between cT1 and cT2a. Patients must have had a prostate multiparametric MRI or undergo this exam within the first 3 months of the study. Patients must be fit for definitive treatment and have availability of biopsy material for diagnostic review.

### Exclusion criteria

Clinical contraindication to prostatectomy or radiotherapy, previous treatment with radical therapies or hormonal therapy, low probability of 10-year survival and biopsy pathology with intraductal carcinoma or cribriform pattern.

### Study procedures and process for obtaining data

**Fig. 1:**
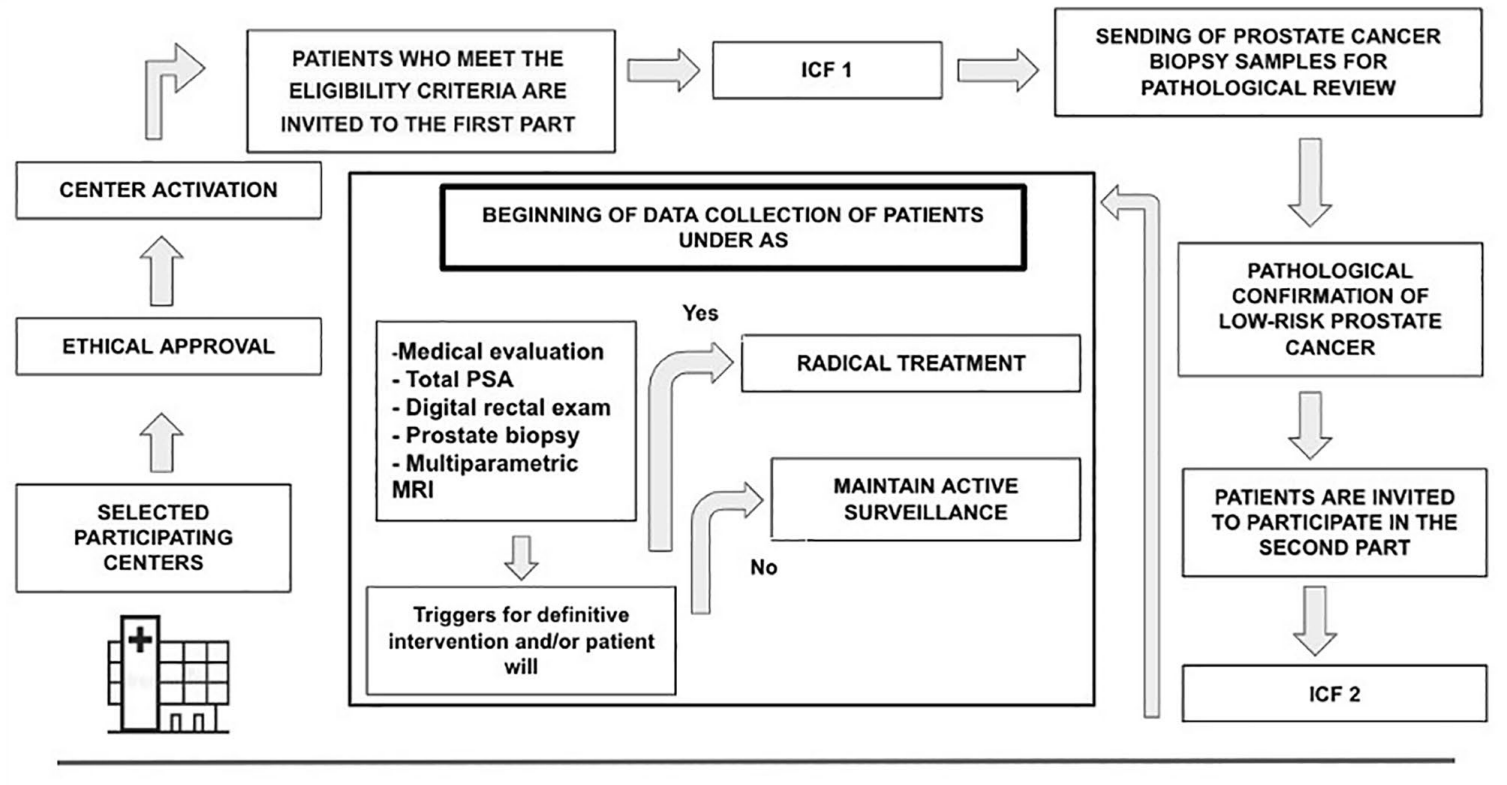
Schematic design of recruitment, approval and start of study collections

Patients who meet the eligibility criteria will be invited to participate and to sign Informed Consent Form 1 (ICF 1). The first part of the study consists of sending prostate biopsy specimens for pathological review to confirm the low-risk diagnosis.***(***Fig. 1***)*** The archived paraffin blocks and slides from prostate biopsy will be sent through a specialized company. The analysis will be performed by a pathological laboratory, and if the results do not show high-risk upgrading, patients will be invited to participate in the second part of the study, in which follow-up data will be collected after patients consent and sign ICF 2. The AS follow-up will be carried out by the local assistant medical team with the guidance of an AS protocol for surveillance (Supplemental 1). All medical decisions will be at the discretion of the assistant team.

### Data collection

Data collection will be carried out individually by the research team of the participating centres. The sociodemographic variables collected will include age, gender, race/skin colour and last month’s family income. Lifetime smoking and alcohol use habits will be recorded. Family history for cancer will be collected, including degree of relationship and age at diagnosis.

Data related to the AS follow-up will be collected, including adherence to the procedures scheduled, data from medical evaluations including symptoms, clinical examination, Charlson Comorbidity Index [15, 16] and total PSA value, PSA density, clinical staging according to the 8th edition of the TNM [17] and data from quality of life questionnaires collected during the study. Multiparametric MRI data and radiological review information from images will be collected, including PI RADS score, number, and localization of suspicious lesions.

Data from prostate biopsies and pathological review will be collected, such as the number and percentage of compromised fragments, location, Gleason score, among others and immunohistochemical markers. Additionally, complications related to monitoring will be recorded, including prostate biopsy complications such as bleeding or infection.

The data collected will be recorded in source documents, such as worksheets and electronic medical records. Then, the data will be entered in the electronic case report form on Research Electronic Data Capture (REDCap). The medical records will be accessed in a restricted way, and confidentiality will be strictly observed by the research team.

### Outcomes

The biopsy pathological reclassification rate as 12 months is the main outcome. Secondary outcomes included the treatment-free survival rate, overall survival rate, cancer-specific mortality rate, metastasis-free survival rate and rate of complications of prostate biopsies.

### Active surveillance protocol

The study AS protocol was designed by the Uro-Oncology Group of the Hospital Moinhos de Vento using the main internationally validated AS protocols as references, such as the Prostate Cancer Research International Active Surveillance (PRIAS) [18]. Our goal with the protocol is to standardize the follow-up of the cohort so that it can serve as a guide for patient management. The protocol describes the inclusion criteria, monitoring schedule and triggers for biopsy and definitive intervention.

### Analysis of prostate biopsy samples

A pathological review of the prostate biopsies will be performed to confirm the low-risk diagnosis. This analysis will be performed in a central pathology laboratory by a specialized uro-oncology pathologist through anatomopathological (AP) and immunohistochemistry (IHC) examination. The AP exam is performed through histological slides stained with haematoxylin and eosin. The architectural arrangement, cytological pattern, presence of perineural permeation, and vascular invasion, among other issues, are evaluated.

IHC exam includes biomarkers related to prostate cancer. A panel with three antibodies will be used: racemase (positive in most neoplastic acini), p63 (whose expression is not identified in neoplastic acini due to the loss of the basal layer), high molecular weight cytokeratin (also lost in neoplasms) and other biomarkers related to prostate cancer, such as PTEN (tensin homologous phosphatase), which may be complementary. The biological samples will be stored in the central laboratory until the end of the study, when they will be returned to the participants.

### Economic impact

In addition to the individual benefits for patients, the AS strategy also generates cost savings, as already demonstrated in economic studies [19, 20].

An assessment of the economic impact of implementing the AS strategy will be carried out by comparing the costs related to this treatment with the costs of immediate treatments, such as radiotherapy and prostatectomy, in patients with low-risk prostate cancer. For this, in addition to mapping the resources allocated to patients in the prospective AS cohort, a retrospective collection of medical records will also be carried out to assess the resources allocated to patients who underwent prostatectomy or radiotherapy from January 2013 to January 2022. Both arms of this substudy will involve considering the costs of medical evaluation, exams, procedures and hospitalizations related to the follow-up of prostate cancer.

### Perceptions of urologists regarding AS

In this study, we will assess the perception of urologists at the participating centres regarding the indications of AS prostate cancer patients in the public health system. For this, each urologist will sign an ICF and answer an online questionnaire at the beginning and end of the study.

### Data analysis and statistics

Descriptive analysis will be used to characterize the study population, with categorical variables presented as absolute (n) and relative (%) frequencies, variables with normal distribution presented as the mean and standard deviation, and variables with asymmetric distribution presented as the median and interquartile range.

We will examine data normality using the Shapiro‒Wilk normality test. The Chi-square test will be applied to detect differences between outcomes and predictor variables with a statistical significance level of 0.05 for all comparisons. Nonparametric tests will be used when appropriate.

Survival analysis will be used to analyse treatment-free survival, overall survival, cause-specific survival and metastasis-free survival. The results will be presented by graphing the estimated survival function and its respective 95% confidence interval (CI).

Budget impact analysis (BIA) will be conducted according to the International Society for Pharmacoeconomics and Outcomes Research (ISPOR) [21] guidelines and Canadian guidelines.

### Sampling and sample size

Considering the rate of pathological reclassification of patients on AS as the main outcome, the estimate for the sample size was 200 patients, using a significance level of 5%, an estimate for the rate of pathological reclassification in 12 months of 24% [18] and an acceptable difference of 10%, to obtain a sample power of more than 80%.

The sample size of 454 medical records for BIA was calculated using the estimate that approximately 30–40% [22] of patients do not need to undergo interventional treatment, with a power of 80%, a significance level of 5% with an acceptable difference of 5% and a design effect of 1.3. Considering that from these records it is possible to have a percentage of missing data, 10% was added, resulting in a final number of 500 reviewed records, approximately.

### Monitoring

Monitoring will be carried out remotely by the coordinating centre. Monitoring is expected to occur on a quarterly basis for each centre participating in the study. In this stage, we will analyse data integrity, verifying whether the data are attributable, readable, contemporary, original and accurate in relation to the source documents. Data reports will be automatically generated using R software, enabling simultaneous monitoring of any missing and inconsistent data.

## Discussion

This is the first Brazilian prospective study of patients with prostate cancer under AS. The study will include centres that are part of the public health system in which many patients are not offered the AS strategy due to insecurity regarding the accuracy of the exams and adherence of patients to monitoring. Data also show that discrepancies in the Gleason score usually occur in approximately 14.7% of biopsy revisions [23]. This study includes a pathological review of all prostate biopsies and a review of mpMRI images. The outcomes of AS will be quantified to assess the applicability of this treatment. The study will also involve evaluating the perception of urologists regarding AS in the public health system and assessing the main problems, which may help health managers plan future improvements.

Brazil is a large country of great diversity and a mixed race population, and this is, to our knowledge, one of the largest AS study cohort with a majority of nonwhite patients. AA men have worse prostate cancer outcomes, which leads to uncertainties in the indication of AS [24]. As large prospective studies have included few AA men, it is generally reported that more prospective observational studies are required before definitive conclusions can be reached [25]. We aim to help understand the clinical outcomes of AS in populations of different races and socioeconomic statuses.

The study has some limitations. Initially, it will have a short follow-up time, but we will seek to extend the duration of the study to obtain long-term outcomes. The Brazilian population is large and involves great diversity between regions, but the study will seek to balance the inclusion of patients. The study will mainly include patients who exclusively use the public health system, but this group represents the majority of the Brazilian population.

A strength of the study is the dissemination of knowledge about AS, a practice considered standard of care in low-risk prostate cancer, which may help to preserve the quality of life of Brazilian men. The data gathered by the study can be used for future investigations in the Brazilian population. Economic impact evaluation can help guide public policies in the country. Important data will be collected on the sociodemographic profile of the population and on the treatment profile currently being applied in the public health system.

### Electronic supplementary material

Below is the link to the electronic supplementary material.

Supplementary Material 1

## Data Availability

Not applicable.

## Abbreviations

AS: Active surveillance
PSA: Prostate-specific antigen
DRE: Digital rectal examination
mpMRI: Multiparametric MRI
AA: African American
ICF: Informed consent form
CCI: Charlson comorbidity index
PRIAS: Prostate Cancer Research International Active Surveillance
IHC: Immunohistochemistry
PTEN: Tensin homologous phosphatase
CI: Confidence interval
CA: Caucasian American
