# Supplementary material for: The Brazilian national prospective active surveillance (AS) cohort of patients with low-risk prostate cancer in the public health system: vigiaSUS study protocol
Source: BMC Urol. 2023 Dec 11;23:208. doi: 10.1186/s12894-023-01380-w (PMC10714582; doi:10.1186/s12894-023-01380-w)
Supplement: Supplementary file 1 — Supplementary Material 1 [file 12894_2023_1380_MOESM1_ESM.docx]

**Supplemental 1**

ACTIVE SURVEILLANCE PROTOCOL IN LOW-RISK PROSTATE CANCER

Guideline for the study “**The Brazilian national prospective active surveillance (AS) cohort of patients with low-risk prostate cancer in the public health system: vigiaSUS study protocol**”

Developed by the Uro-Oncology Group of Hospital Moinhos de Vento

Version 4: 28/10/2023

**Inclusion criteria**

1 Pathological diagnosis of prostate adenocarcinoma

2 PSA less than or equal to 10 ng/ml*

3 Clinical staging less than or equal to cT2a

4 Gleason score below or equal to 6 (3+3)

5 Prostatic biopsy with at least 12 cores

6 Adequate clinical condition for definitive treatment

7 Performed or planned multiparametric prostate MRI

* If PSA is above 10 ng/ml, conditions such as prostatic hyperplasia, prostatitis, and increased PSA after biopsy may be considered and allow inclusion of the patient in the protocol.

**Exclusion criteria**

1 Clinical contraindication to prostatectomy or radiotherapy procedures

2 Life expectancy below 10 years

3 Previous treatments with hormone therapy, prostatectomy or radiotherapy

4 Presence of intraductal or cribriform histology in biopsy

**Monitoring schedule** (Table S1)

**Table S1.** Monitoring schedule

| **Year** | **1** | | | | | **2** | | **3** | | **4** | | **5** | | **6** | |
| --- | --- | --- | --- | --- | --- | --- | --- | --- | --- | --- | --- | --- | --- | --- | --- |
| **Months** | **0** | **3** | **6** | **9** | **12** | **18** | **24** | **30** | **36** | **42** | **48** | **54** | **60** | **66** | **72** |
| Clinical evaluation | x |  | x |  | x | x | x | x | x | x | x | x | x | x | x |
| Total PSA | x |  | x |  | x | x | x | x | x | x | x | x | x | x | x |
| Digital rectal exam | x |  | x |  | x | x | x | x | x | x | x | x | x | x | x |
| TRUS  biopsy | x |  |  |  | x |  |  |  | x |  |  |  | x |  |  |
| mpMRI +/- guided biopsy |  | x |  |  |  |  | x |  |  |  | x |  |  |  | x |
| Quality of life | x |  |  |  | x |  | x |  | x |  | x |  | x |  | x |

*Month 0 is defined as the date on which the anatomopathological and/or immunohistochemical report based on the diagnosis of low-risk neoplasia

*If the patient enters the study after month 0, the quality of life questionnaires must be administered when the patient enters the study and subsequently repeated annually.

*The mpMRI with or without biopsy in cases that do not have the exam when entering the study must be performed within 3 months of the patient's entry into the study and subsequently in the schedule periods. In cases where the baseline MRI shows a lesion that has high probability of clinically significant cancer (PIRADS > or =3) or signs of a locally advanced tumor (T3), a guided biopsy is indicated.

**Evaluation of eligibility criteria**

Patients diagnosed with prostate cancer and candidates for the active surveillance strategy should have the following criteria evaluated.

- Assessment of life expectancy using the Charlson Comorbidity index (ICC)

- Description of TRUS Prostate Biopsy including number of cores

- Prostate biopsy pathology report of adenocarcinoma including Gleason score, description of compromised fragments and percentage in which the fragments are compromised

- Recent total PSA

- PSA density

- Digital rectal examination with clinical staging

- Evaluation of conditions that may contraindicate radical prostatectomy and radiotherapy treatments

- If a mpMRI was performed, description of the result and whether a guided biopsy was performed

- Quality of life questionnaires: EQ-5D-5 L and EPIC-CP and anxiety: GAD-7

**Biopsy triggers**

Triggers to anticipate a prostate biopsy include clinical and laboratory alterations that suggest a change in tumour grade or disease progression. Indications for early prostate biopsies are biochemical PSA progression and mpMRI changes.

***Biochemical progression***

- If the total PSA is greater than 10 ng/ml

- If the PSADT calculation is less than or equal to 3 years, evaluated after 12 months of follow-up using the Memorial Sloan Kettering Cancer Center calculator

***Progression by imaging exam***

- The first mpMRI with a PI-RADS score greater than or equal to 3 indicates the need for guided biopsy.

- If the second mpMRI exam presents a PRECISE classification greater than 3, guided biopsy is indicated.

**Triggers for definitive intervention**

Triggers for definitive intervention by radical prostatectomy or radiotherapy include:

- Clinical progression (>cT3)

- Pathological reclassification in biopsy with increased tumour grade with Gleason score greater than 6 (3+3).

- Patient dissatisfaction with the VA strategy

**Life expectancy assessment**

During each clinical evaluation, a reassessment of comorbidities and relevant clinical events should be performed. Life expectancy should be estimated by age and BCI. If the probability of 10-year survival is low, the patient has an indication for the "watchful waiting" strategy and will leave the protocol.

The life estimate will be evaluated by the ICC.

**Evaluation of metastatic disease**

The investigation of metastatic disease is at the discretion of the attending physician, considering suspicious symptoms and PSA increase, mainly above 10 ng/ml.
